# Species-Specific Expression of Full-Length and Alternatively Spliced Variant Forms of CDK5RAP2

**DOI:** 10.1371/journal.pone.0142577

**Published:** 2015-11-09

**Authors:** John S. Y. Park, Marie-Katrina Lee, SungMyung Kang, Yan Jin, Songbin Fu, Jesusa L. Rosales, Ki-Young Lee

**Affiliations:** 1 Department of Cell Biology and Anatomy, Southern Alberta Cancer Research and Hotchkiss Brain Institutes, University of Calgary, Calgary, Alberta, Canada; 2 Laboratory of Medical Genetics, Harbin Medical University, Harbin, China; 3 Department of Biochemistry and Molecular Biology, Southern Alberta Cancer Research and Hotchkiss Brain Institutes, University of Calgary, Calgary, Alberta, Canada; The Hong Kong University of Science and Technology, HONG KONG

## Abstract

CDK5RAP2 is one of the primary microcephaly genes that are associated with reduced brain size and mental retardation. We have previously shown that human CDK5RAP2 exists as a full-length form (hCDK5RAP2) or an alternatively spliced variant form (hCDK5RAP2-V1) that is lacking exon 32. The equivalent of hCDK5RAP2-V1 has been reported in rat and mouse but the presence of full-length equivalent hCDK5RAP2 in rat and mouse has not been examined. Here, we demonstrate that rat expresses both a full length and an alternatively spliced variant form of CDK5RAP2 that are equivalent to our previously reported hCDK5RAP2 and hCDK5RAP2-V1, repectively. However, mouse expresses only one form of CDK5RAP2 that is equivalent to the human and rat alternatively spliced variant forms. Knowledge of this expression of different forms of CDK5RAP2 in human, rat and mouse is essential in selecting the appropriate model for studies of CDK5RAP2 and primary microcephaly but our findings further indicate the evolutionary divergence of mouse from the human and rat species.

## Introduction

The autosomal recessive primary microcephaly (MCPH) is a rare neurodevelopmental disorder that is associated with reduced brain size, particularly of the cerebral cortex, and nonprogressive mental retardation [1, 2]. The premise is that small brain size results from asymmetric division of neuronal progenitor cells [3], leading to the notion that MCPH is a primary disorder of neurogenic mitosis [4]. The role of MCPH genes in neurogenic mitosis is unclear but proposed to be involved in the control of neural progenitor pool expansion or switch from symmetric to asymmetric cell division [4].

One of the twelve MCPH genes [5–7] is ***MCPH3*** which encodes cyclin-dependent kinase 5 (CDK5) [8, 9] regulatory subunit-associated protein 2 (CDK5RAP2) [10] that was first described [11]. The human CDK5RAP2 gene (***hCDK5RAP2***) consists of 38 exons, encoding a 1893 amino acid residue protein that is composed of a CM1 domain (residues 58–90)[12] that binds γ-tubulin complexes, two structural maintenance of chromosome (SMC) domains (one each in the N- and C- terminals, corresponding to residues 137–470 and 1399–1646, respectively)[1], EB1 binding region (residues 926–1208)[13], a p35 interacting domain (residues 1682–1827)[11, 14, 15], and a pericentrin binding CM2 domain (residues 1726–1893)[16].

A number of discrete mutations in the CDK5RAP2 gene have been reported. For example, two in Pakistani families (n = 10), one in a Somali patient (n = 1), and one in two (n = 2) patients of European descent. In the northern Pakistani microcephaly pedigrees [15, 17], the first pedigree mutation (246T→A, Y82X; n = 8) generates a truncated 81 amino acid peptide that lacks a portion of the SM1 binding motif, the two SMC motifs, the EB1 binding region, and the p35- and pericentrin-binding regions. The second pedigree mutation (IVS26-15A→G, R1334SfsX5, n = 2) in northern Pakistani microcephaly pedigrees [15, 17], on the other hand, results in a truncated 1338 amino acid protein that lacks the EB1 binding region, the C-terminal SMC and the p35- and pericentrin-binding motifs. A third pedigree mutation (4441C→T) from two (n = 2) patients of European descent [17] results in a truncated 1481 amino acid protein that lacks part of C-terminal SMC and the p35- and pericentrin-binding regions motifs. A fourth mutation (700G→T) from an individual (n = 1) of Somali descent [18] encodes a truncated 234 amino acid protein that lacks a portion of the CM1 binding motif, the EB1 binding region, and the p35- and pericentrin-binding regions. Together, these findings indicate that the loss of the C-terminal SMC and p35- and pericentrin-binding motifs are sufficient to cause CDK5RAP2-associated primary microcephaly.

In an effort to identify an appropriate model to further investigate CDK5RAP2 and primary microcephaly, we sought to perform a comparative analysis of CDK5RAP2 expression in rat and mouse. We determined that in rat, a full length and an alternatively spliced variant forms of CDK5RAP2 exist (rCDK5RAP2 and rCDK5RAP2-V1, respectively). These two forms of rat CDK5RAP2 are equivalent to the human full length and alternatively spliced variant forms of CDK5RAP2 that we identified previously (hCDK5RAP2 and hCDK5RAP2-V1, repectively). Mouse, on the other hand, expresses only one form of CDK5RAP2 that is equivalent to the human and rat alternatively spliced variant forms. Sequence details and tissue expression of these CDK5RAP2 forms are presented.

## Materials and Methods

### Animals

All animal works have been conducted according to the protocol (AC13-0109_MOD2) approved by the Health Sciences Animal Care Committee of the University of Calgary. Rats were housed at the University of Calgary Animal Resource Centre which conforms to the CCAC standards, allowed unlimited access to food [Prolab RMH2500 rodent diet (Purina Lab)] and water, and cared daily by animal resource Centre. Rats were euthanized by IP injection of sodium pentobarbital (100 mg/kg) followed by cervical dislocation according to SOP:E1 to minimize potential suffering prior to harvesting tissues.

### Analysis of CDK5RAP2 genomic sequences in rat and mouse

The UCSC Genome Browser on Rat, Mar 2012 (RGSC 5.0/rn5) Assembly (rat chromosome 5q31 minus strand from nucleotide 90474208 to nucleotide 90641322), the UCSC Genome Browser on Mouse, Dec 2011 (GRCm38/mm10) Assembly (mouse chromosome 4qC2 minus strand from nucleotide 70223024 to nucleotide 70410367), and the UCSC Genome Browser on Human Feb 2009 (GRCh37/hg19) Assembly (human chromosome 9q33.2 minus strand from nucleotide 123151147 to nucleotide 123342437) from the GenBank database were adapted for analysis.

### Sequence alignment of CDK5RAP2 in rat, mouse and human, and analysis of the primary structure of rat and mouse CDK5RAP2

The sequences of rCDK5RAP2, rCDK5RAP2-V1, mCDK5RAP2, hCDK5RAP2 and hCDK5RAP2-V1 were aligned using the CLUSTAL 2.1 multiple sequence alignment software. rCDK5RAP2 (genbank accession no. JX524852), rCDK5RAP2-V1 (genbank accession no. NM_173134), mCDK5RAP2 (genbank accession no. NM_145990), hCDK5RAP2 (genbank accession no. NM_018249) and hCDK5RAP2-V1 (genbank accession no. NM_001011649) were used. Analysis of the primary structure of rCDK5RAP2, rCDK5RAP2-V1 and mCDK5RAP2, and designation of amino acid numbers for the respective domains were based on sequence alignment with hCDK5RAP2 using CLUSTAL 2.1 multiple sequence alignment software.

### RT-PCR analysis

cDNA panel of Sprague-Dawley rats (ages 8–12 weeks) and BALB/c mice (age 8–12 weeks) and mouse embryos from Swiss Webster/NIH mice purchased from Clontech Labs. Expression of rat and mouse cdk5RAP2 was determined by RT-PCR using rat and mouse tissue cDNAs as templates. For rcdk5RAP2 and rcdk5RAP2-v1 PCR, the forward primer TATGAGAAGCTGGCCGAGGAG and the reverse primer TAGGTGCCATTGCCTGGGTAG were used. RPL13A was used as an internal control: the forward primer was TCCCTCCACCCTATGACAAGAA and the reverse primer was GCTGTCACTGCCTGGTACTTCC. For mCDK5RAP2 PCR, the forward primer CCAAATGGAGCTCAAGGTGTATG and the reverse primer CTGCTCACAGTGAAGGGAACACT were used. S16 was used as an internal control: the forward primer was AGGAGCGATTTGCTGGTGTGGA and the reverse primer was GCTACCAGGGCCTTTGAGATGGA were used. No template was used for the negative control. Levels of rcdk5RAP2, rcdk5RAP2-v1 and mCDk5rap2 transcripts were measured by densitometric analysis using the NIH Image J software.

### DNA sequencing

The PCR products described above were gel purified using Qiaquick Gel Extraction Kit (Qiagen). Sequencing was performed following Sanger’s method at the University of Calgary Core DNA Services using Applied Biosystems 3730XL 96 Capillary Sequencer.

### Northern blot analysis

Northern blot analysis was performed as described previously [19]. Briefly, total RNA (2 μg each) was isolated from mouse spleen and testis, and rat spleen. Samples were resolved by 1.5% formaldehyde agarose gel electrophoresis, immobilized onto a nylon membrane, pre-hybridized at 42°C overnight, and hybridized at 42°C overnight with two specific probes. The 223 bp probe to verify the presence of full-length mCDK5RAP2 was generated by PCR using mouse testis cDNAs as template and using the forward and reverse primers, GGTGCAGGAAGA GGCGAAGT and AAATAGATCAAATGAATTGTCGCTG, respectively. The 102 bp probe to verify the absence of exon 32 that is found in full-length hCDK5RAP2 and in full-length rCDK5RAP2 was generated by PCR using mouse tail genomic DNAs as template and using the forward and reverse primers, AGAAAGGAGCATTCAAAC TAACAG) and ACAGCCAAAGTGAGGGCCCC, respectively. Initial PCR products were generated in a 50 μl reaction mixture containing 1x PCR buffer (Invitrogen), 2 mM MgCl_2_, 0.4 mM of dNTPs, 0.4 μl Taq polymerase (Invitrogen) and 1 μM each of the primers, then amplified using 33 cycles at 94°C for 30 sec, 52°C for 60 sec, and 72°C for 60 sec. The PCR products were then gel purified using Qiaquick Gel Extraction Kit (Qiagen) and confirmed by sequencing. The [α-^32^P] labeled probes were then generated using the initial PCR product as template in a 50 μl reaction mixture containing 1x PCR buffer (Invitrogen), 2 mM MgCl_2_, 0.4 mM of each dATP, dGTP and dTTP, 0.4 μl Taq polymerase (Invitrogen) and 1 μM each of the reverse primers + 2.5 μl of [α-^32^P] dCTP (3000 Ci/mmole; PerkinElmer), then amplified using 15 cycles at 94°C for 30 sec, 52°C for 60 sec, and 72°C for 60 sec.

## Results and Discussion

Previously, we have demonstrated that human CDK5RAP2 (hCDK5RAP2) exists in at least two forms: full-length form (hCDK5RAP2) and an alternatively spliced variant form (hCDK5RAP2-V1), that is lacking exon 32 (237 nucleotides) of the full-length hCDK5RAP2 [14]. In addition, we showed differential tissue distribution of hCDK5RAP2 and hCDK5RAP2-V1. For example, compared to hCDK5RAP2, we demonstrated significantly decreased expression of hCDK5RAP2-V1 in kidney, lung and placenta. We also showed that hCDK5RAP2-V1 is barely detectable in brain. These findings suggest distinct roles of hCDK5RAP2 and hCDK5RAP2-V1 *in vivo*.

**Fig 1:**
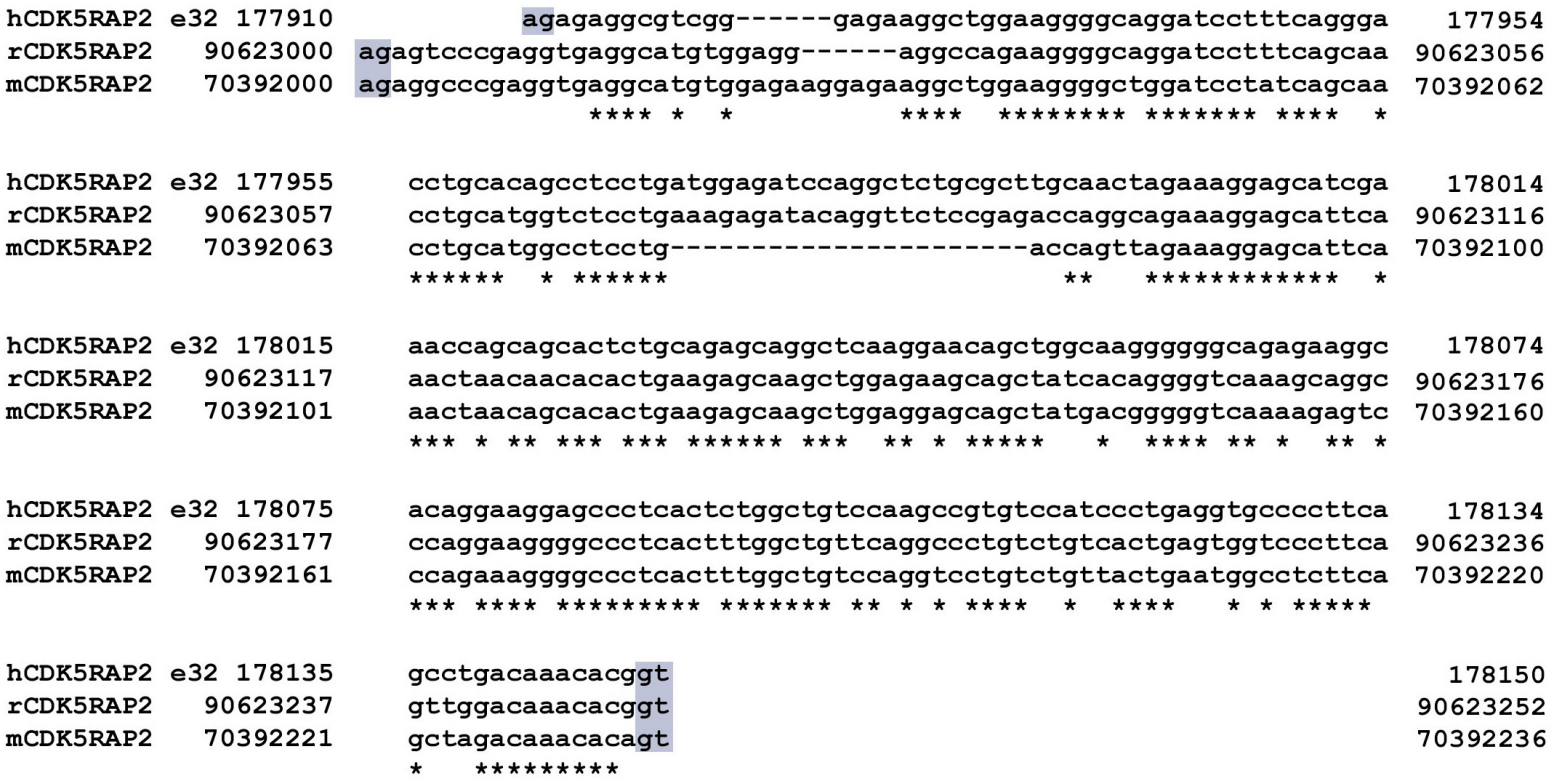
Genomic CDK5RAP2 sequence alignment reveals the existence of a comparable rCDK5RAP2 and mCDK5RAP2 exon 32. The identical nucleotide sequences in rCDK5RAP2, mCDK5RAP2 and hCDK5RAP2 are shown (asterisk). The AG-GT exon-intron boundary sequences (shaded in grey) in genomic rCDK5RAP2, mCDK5RAP2 and hCDK5RAP2 sequences were also shown.

Soon after our report, an account of the existence of a hCDK5RAP2-V1 equivalent in rat and mouse was presented [20] but the existence of a full-length rat and mouse CDK5RAP2 (rCDK5RAP2 and mCDK5RAP2, respectively) in the non-redundant nucleotide or protein databases was not demonstrated. Interestingly, however, our analysis of CDK5RAP2 genomic sequences in the GenBank database revealed the existence of a comparable rCDK5RAP2 and mCDK5RAP2 exon 32 sequences in rat and mouse with the AG-GT exon-intron boundary sequences (Fig 1).

**Fig 2:**
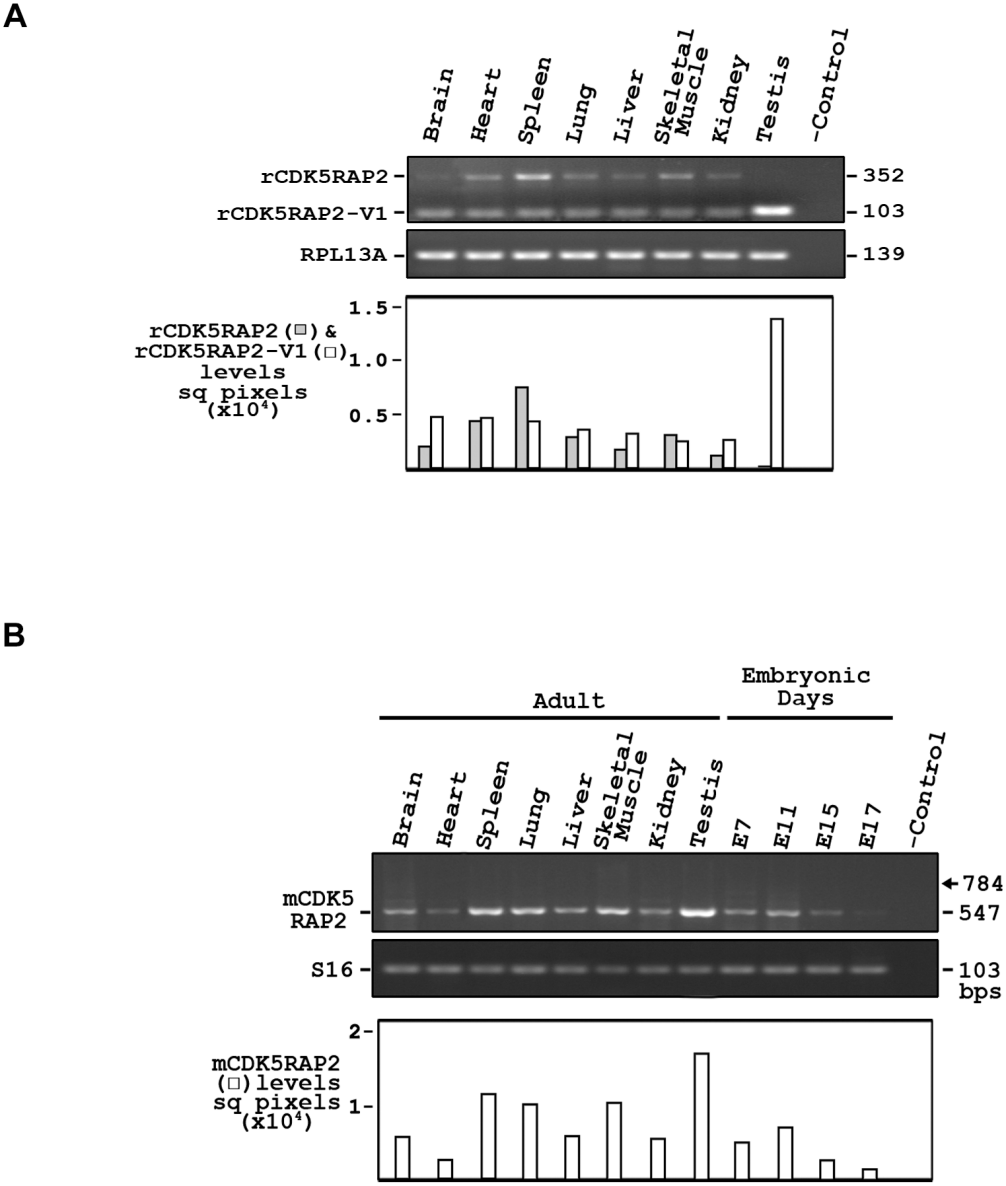
A. Expression analysis of rat cdk5RAP2. Tissue distribution of rcdk5RAP2 and rcdk5RAP2-v1 transcripts as determined by RT-PCR. Rat tissue cDNAs were used as templates. No template was used for the negative control. The bottom panels show the levels of rcdk5RAP2 and rcdk5RAP2-v1 transcripts as measured by densitometry using the NIH Image J software. Data represent one of three independent experiments (n = 3). B. Expression analysis of mouse cdk5RAP2. Tissue distribution of mCDk5rap2 transcripts as determined by RT-PCR. Mouse tissue cDNAs were used as templates. No template was used for the negative control. The bottom panels show the levels of mCDk5rap2 (right panel) transcripts as measured by densitometry using the NIH Image J software. Data represent one of three independent experiments (n = 3).

We therefore investigated whether rat and mouse express the full-length CDK5RAP2 forms that are equivalent to hCDK5RAP2 as well as alternatively spliced variant forms (rCDK5RAP2-V1 and mCDK5RAP2-V1) that are equivalent to hCDK5RAP2-V1. As shown in Fig 2A, the presence of both full-length rCDK5RAP2 and alternatively spliced rCDK5RAP2-V1 transcripts, corresponding to 352 bp and 103 bp PCR fragments, respectively, was analyzed in various rat tissues by RT-PCR analysis using forward and reverse primers (see Materials and Method section) spanning human exon 32-like region.

Our data indicate that rat expresses both full-length CDK5RAP2 (rcdk5RAP2) and an alternatively spliced variant form 1 (rcdk5RAP2-v1) that are equivalent to our previously reported hCDK5RAP2 and hCDK5RAP2-V1 [14], respectively. Both rcdk5RAP2 and rcdk5RAP2-v1 are expressed at almost similar levels in most tissues, including in the heart, lung, liver, skeletal muscle and kidney. However, it is quite interesting that although rcdk5RAP2-v1 is clearly present in brain, rcdk5RAP2 is minimally expressed. Furthermore, in testis, rCDK5RAP2 is absent while rCDK5RAP2-V1 is highly expressed. These striking differences suggest a crucial role for rCDK5RAP2-V1 in both the brain and testis.

We then analyzed various mouse tissues for the presence of both full-length mCDK5RAP2 and alternatively spliced mCDK5RAP2-V1 transcripts, which correspond to 784 bp and 547 bp PCR fragments, respectively, by RT-PCR analysis using appropriate forward and reverse primers (see Materials and Method section) spanning human exon 32-like region. We found that the anticipated 784 bp fragment, corresponding to full-length hCDK5RAP2 and rCDK5RAP2, was not detected in all tissues examined (Fig 2B, arrow). Instead, only a 547 bp mCDk5rap2 fragment, corresponding to the alternatively spliced hCDK5RAP2-V1 and rCDK5RAP2-V1 was detected, indicating that mouse does not expresses the full-length equivalent of hCDK5RAP2 and rCDK5RAP2, and that the full-length mCDK5RAP2 corresponds to the alternatively spliced hCDK5RAP2-V1 and rCDK5RAP2-V1. As with rCDK5RAP2-V1, mCDK5RAP2 is highly expressed in testis. We also observed that the expression of mCDK5RAP2 is regulated during development with higher levels at embryonic day 7 (E7) and E11, decreased expression at E15 and significantly reduced expression at E17. These findings are consistent with those reported previously [21] in mouse brain where expression peaks of CDK5RAP2 were observed during periods of active neurogenesis in the developing neocortex. The differential tissue distribution of the different forms of CDK5RAP2 in rat and mouse as well as the developmental regulation of mCDK5RAP2 expression is intriguing and further suggests distinct roles *in vivo*. Certainly, these issues may be worth investigating.

**Fig 3:**
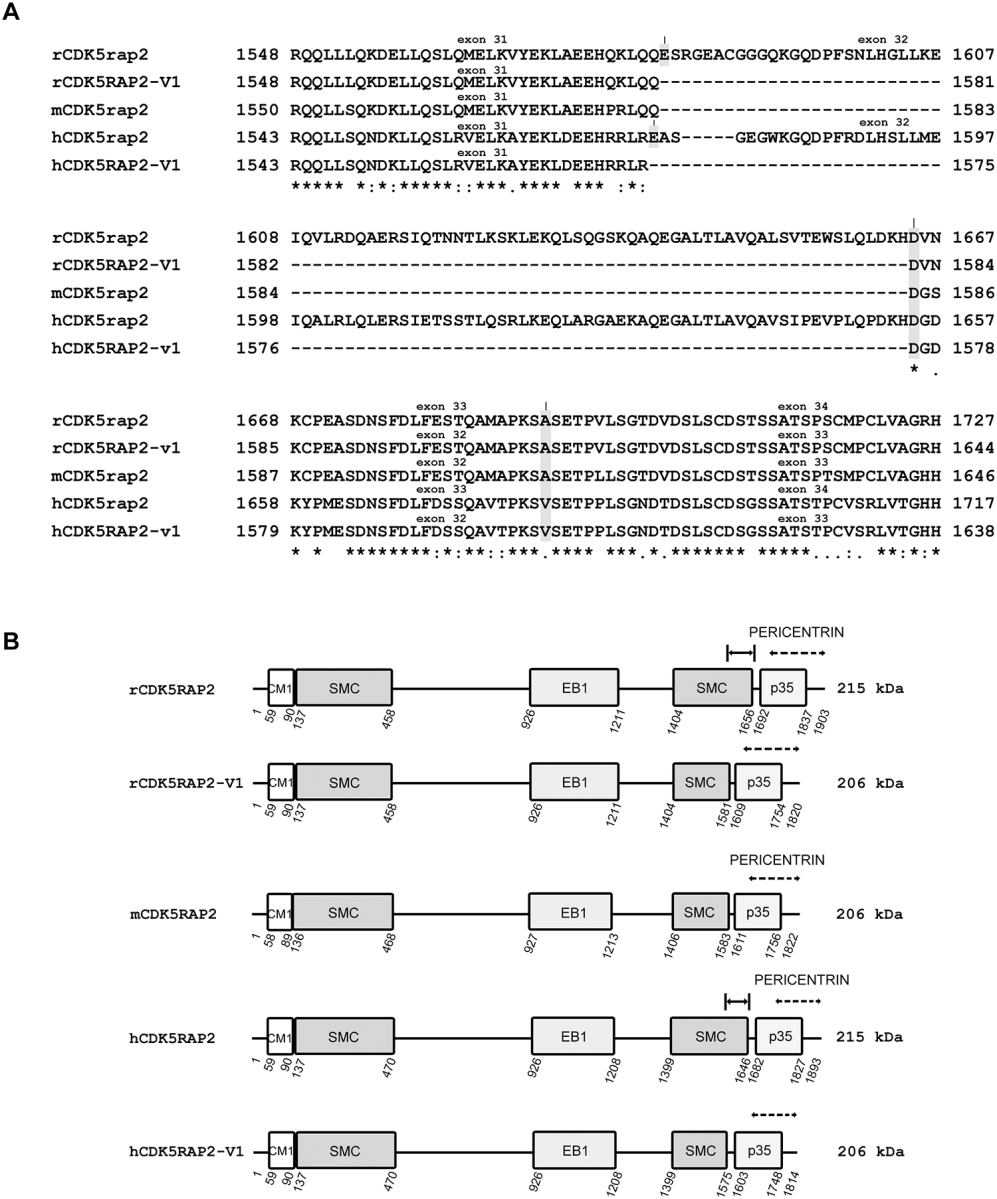
A. The equivalent of exon 32 in full-length rat cdk5RAP2 (rcdk5RAP2) is absent in the alternatively spliced variant form of rat cdk5RAP2 (rcdk5RAP2-v1) and in the mouse full-length cdk5RAP2 (mCDk5rap2). Sequences of rCDK5RAP2, rCDK5RAP2-V1, mCDK5RAP2, hCDK5RAP2 and hCDK5RAP2-V1 are aligned and amino acid numbers are indicated on either side. The identical sequences in rCDK5RAP2, rCDK5RAP2-V1, mCDK5RAP2, hCDK5RAP2 and hCDK5RAP2-V1 are shown. The broken lines correspond to the amino acid sequence missing in rcdk5RAP2-v1, mCDK5RAP2, hCDK5RAP2 and hCDK5RAP2-V1. The codon (GAG) encoding glutamic acid (E, shaded in grey) in rCDK5RAP2 and hCDK5RAP2 is generated from the combination of nucleotides (G-AG) between exon 31 and exon 32. The codon (GAT) encoding aspartic acid (D, shaded in grey) in rCDK5RAP2, rCDK5RAP2-V1, mCDK5RAP2, hCDK5RAP2 and hCDK5RAP2-V1 is generated from the combination of nucleotides (G-AT) between exon 32 and exon 33 in rCDK5RAP2 and hCDK5RAP2, and exon 31 and exon 32 in rCDK5RAP2-V1, mCDK5RAP2 and hCDK5RAP2-V1, respectively. B. Primary structures of rCDK5RAP2, rCDK5RAP2-V1 and mCDK5RAP2. The amino acid numbers for the respective domains were based on sequence alignment with hCDK5RAP2 using CLUSTAL 2.1 multiple sequence alignment software. rCDK5RAP2 encodes 1903 amino acids while rCDK5RAP2-V1 encodes 1820 amino acids lacking 83 amino acids (aa_1582_-aa_1664_) that are found in rCDK5RAP2. The missing amino acids (1582 to 1664) in rCDK5RAP2-V1 are also absent in mCDK5RAP2, which encodes 1822 amino acids. Note that the C-terminal portion of the second SMC domain in rCDK5RAP2 is absent in rCDK5RAP2-V1 and mCDK5RAP2 as well. hCDK5RAP2 encodes 1,893 amino acids (215 kDa) while hCDK5RAP2-V1 encodes 1,814 amino acids (206 kDa) lacking 79 amino acids from amino acid_1,576_ to amino acid_1,654_. hCDK5RAP2 (genbank accession no.NP_060719) and hCDK5RAP2-V1 (genbank accession no. NP_001011649) are shown.

As shown in Fig 3A and 3B, sequencing analysis of the 352 and 103 bp PCR products and subsequent analysis of deduced amino acid sequence confirmed that the full-length rCDK5RAP2 (GenBank, accession number: JX524852) contains exon 32 and encodes 1903 amino acids (215 kDa).

**Fig 4:**
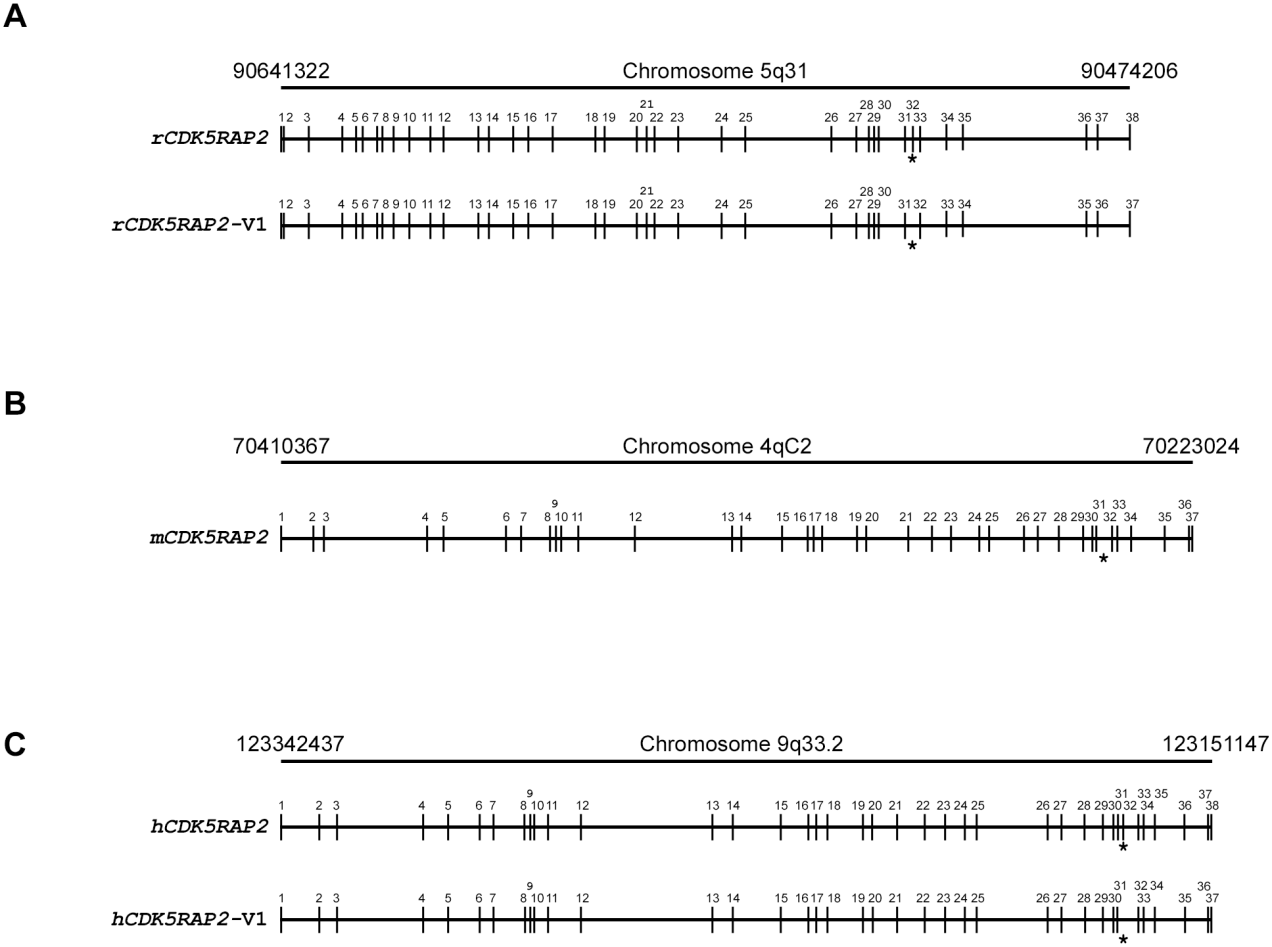
Schematic diagram of CDK5RAP2 gene in rat chromosome 5q31 minus strand from nucleotide 90474208 to nucleotide 90641322 [adapted from UCSC Genome Browser on Rat Mar 2012 (RGSC 5.0/rn5) Assembly], mouse chromosome 4qC2 minus strand from nucleotide 70223024 to nucleotide 70410367 [adapted from UCSC Genome Browser on Mouse Dec 2011 (GRCm38/mm10) Assembly], and human chromosome 9q33.2 minus strand from nucleotide 123151147 to nucleotide 123342437 [adapted from UCSC Genome Browser on Human Feb 2009 (GRCh37/hg19) Assembly. Vertical bars with numbers indicate exons of the full-length rcdk5RAP2, the alternatively spliced variant form 1, rcdk5RAP2-v1, the mouse CDk5rap2 (mCDk5rap2), the full-length human (hcdk5RAP2), and the human alternatively spliced variant form 1 (hcdk5RAP2-v1). Note that the equivalent of exon 32 in full-length rcdk5RAP2 is absent in the alternatively spliced rcdk5RAP2-v1 and the mouse CDk5rap2 and alternatively spliced hcdk5RAP2-v1 (asterisk).

On the other hand, the 103 bp PCR product of the alternatively spliced rCDK5RAP2-V1 encodes 1820 amino acids (206 kDa), and lacks 83 of the amino acids (aa_1582_ to aa_1664_) found in full-length rCDK5RAP2. Sequencing analysis of the 547 bp PCR product of full-length mCDK5RAP2 showed that the amino acid residues (aa_1582_ to aa_1664_) found in rCDK5RAP2 is also absent in full-length mCDK5RAP2 (Fig 3A and 3B). This finding suggests that the full-length mCDK5RAP2 encodes 1822 amino acids (206 kDa) and lacks the exon 32 (noted by asterisk, Fig 4) that is found in full-length hCDK5RAP2 and full-length rCDK5RAP2.

Interestingly, our analysis also revealed that the C-terminal portion of the second SMC domain in full-length rCDK5RAP2 is absent in the alternatively spliced rCDK5RAP2-V1 and in full-length mCDK5RAP2. This occurrence may be linked to the absence of exon 32 (in rCDK5RAP2-V1 and mCDK5RAP2) that spans part of the C-terminal SMC plus 8 amino acid residues after the C-terminal SMC (Fig 3B; indicated by I↔I). Whether this difference may also be involved in the specificity of DNA-protein interaction remains to be determined but could further support our premise that the different forms of CDK5RAP2 have distinct roles *in vivo*.

**Fig 5:**
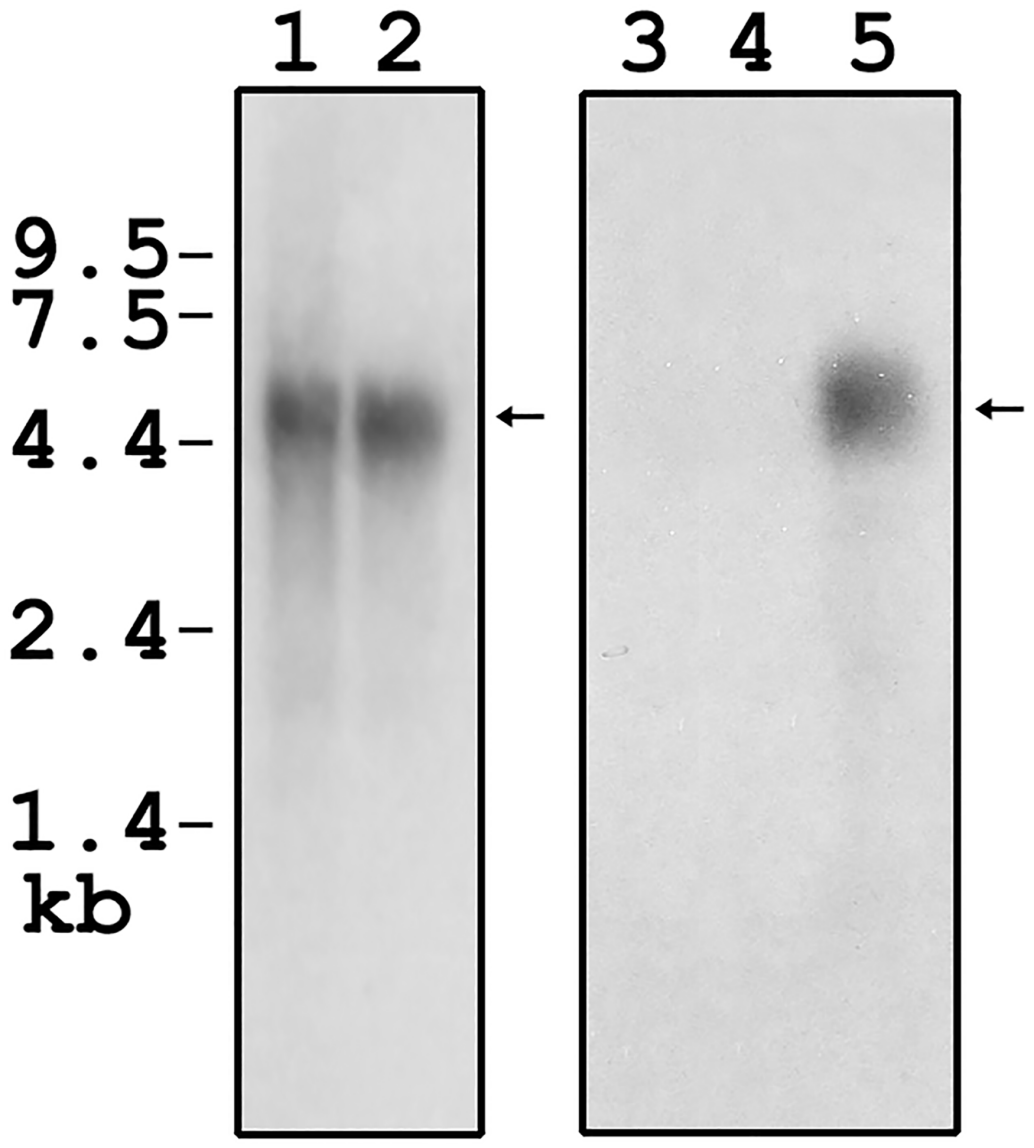
Northern blot analysis of mCDK5RAP2. Analysis was performed as described in Materials and Methods. Lanes 1 (mouse spleen) and 2 (mouse testis) were hybridized with a 223 bp probe that spans exon 31 and exon 32 of mCDK5RAP2. Lanes 3 (mouse spleen), 4 (mouse testis) and 5 (rat speen) were hybridized with a 102 bp probe that spans the exon 32-like mouse gDNA sequence that corresponds to exon 32 in full-length hCDK5RAP2 and full-length rCDK5RAP2. The absence of mRNA in lanes 3 and 4 indicates that the mCDK5RAP2 lacks exon 32 that is found in full-length hCDK5RAP2 and in full-length rCDK5RAP2. Rat spleen RNA was used as positive control (lane 5).

We then performed northern blot analysis to further verify the existence of only one mCDK5RAP2. Fig 5 shows the presence of a ~5.4 kb mCDK5RAP2 mRNA in representative mouse tissues (lane 1, spleen; lane 2, testis), when hybridized with a 223 bp probe that spans exon 31 and exon 32 of mCDK5RAP2. However, no mRNA was detected in either tissue (lane 3, spleen; lane 4, testis) when hybridized with a 102 bp probe that spans the exon 32-like mouse gDNA sequence, which corresponds to exon 32 in the full-length hCDK5RAP2 and full-length rCDK5RAP2. This result indicates the absence of a mouse CDK5RAP2 mRNA that corresponds to full-length hCDK5RAP2 and full-length rCDK5RAP2. This further supports our view that the full-length mCDK5RAP2 encodes 1822 amino acids and lacks the exon 32 that is found in full-length hCDK5RAP2 and full-length rCDK5RAP2. It is possible that lack of full length CDK5RAP2 containing exon 32 or second SMC domain is related to reduced neuronal progenitor cell division and smaller brain size. However, this premise remains to be investigated.

Knowledge of the differential expression of the different forms of CDK5RAP2 in human, rat and mouse is essential when choosing a suitable model for further studies on CDK5RAP2 and primary microcephaly. In addition, from an evolutionary standpoint, the presence of exon 32 in full-length CDK5RAP2 in human and in rat but not in mouse indicates evolutional divergence of mouse from the human and rat species.
